# Supplementary material for: Resolving the full spectrum of human genome variation using Linked-Reads
Source: Genome Res. 2019 Apr;29(4):635–45. doi: 10.1101/gr.234443.118 (PMC6442396; doi:10.1101/gr.234443.118)
Supplement: Supplemental Material [file supp_gr.234443.118_Supplemental_longranger-2.2.zip › longranger-2.2.tar.bz2!/lr2.2-public/lib/stan/cmdstan/stan/doc/api/html/classstan_1_1io_1_1dump__reader.html]

Stan: stan::io::dump\_reader Class Reference


|  |  |
| --- | --- |
|  | Stan  2.5.0  probability, sampling & optimization |


- Main Page
- Related Pages
- Namespaces
- Classes
- Files

- Class List
- Class Index
- Class Hierarchy
- Class Members

All Classes Namespaces Files Functions Variables Typedefs Enumerations Enumerator Friends Macros Pages

- stan
- io
- dump\_reader

Public Member Functions |
List of all members

stan::io::dump\_reader Class Reference

Reads data from S-plus dump format.
More...

`#include <dump.hpp>`

|  |  |
| --- | --- |
| Public Member Functions | |
|  | dump\_reader () |
|  | Construct a reader for standard input. More... |
|  | |
|  | dump\_reader (std::istream &in) |
|  | Construct a reader for the specified input stream. More... |
|  | |
|  | ~dump\_reader () |
|  | Destroy this reader. More... |
|  | |
| std::string | name () |
|  | Return the name of the most recently read variable. More... |
|  | |
| std::vector< size\_t > | dims () |
|  | Return the dimensions of the most recently read variable. More... |
|  | |
| bool | is\_int () |
|  | Checks if the last item read is integer. More... |
|  | |
| std::vector< int > | int\_values () |
|  | Returns the integer values from the last item if the last item read was an integer and the empty vector otherwise. More... |
|  | |
| std::vector< double > | double\_values () |
|  | Returns the floating point values from the last item if the last item read contained floating point values and the empty vector otherwise. More... |
|  | |
| bool | next () |
|  | Read the next value from the input stream, returning `true` if successful and `false` if no further input may be read. More... |
|  | |

## Detailed Description

Reads data from S-plus dump format.

A `dump_reader` parses data from the S-plus dump format, a human-readable ASCII representation of arbitrarily dimensioned arrays of integers and arrays of floating point values.

Stan's dump reader is limited to reading integers, scalars and arrays of arbitrary dimensionality of integers and scalars. It is able to read from a file consisting of a sequence of dumped variables.

There cannot be any `NA` (i.e., undefined) values, because these cannot be represented as `double` values.

The dump reader class follows a standard scanner pattern. The method `next()` is called to scan the next input. The type, dimensions, and values of the input is then available through method calls. Here, the type is either double or integer, and the values are the name of the variable and its array of values. If there is a single value, the dimension array is empty. For a list, the dimension array contains a single entry for the number of values. For an array, the dimensions are the dimensions of the array.

Reads are performed in an "S-compatible" mode whereby a string such as "1" or "-127" denotes and integer, whereas a string such as "1." or "0.9e-5" represents a floating point value.

For dumping, arrays are indexed in last-index major fashion, which corresponds to column-major order for matrices represented as two-dimensional arrays. As a result, the first indices change fastest. For instance, if there is an three-dimensional array `x` with dimensions `[2,2,2]`, then there are 8 values, provided in the order

`[0,0,0]`, `[1,0,0]`, `[0,1,0]`, `[1,1,0]`, `[0,0,1]`, `[1,0,1]`, `[0,1,1]`, `[1,1,1]`.

definitions ::= definition+

definition ::= name ("->" | '=') value optional\_semicolon

name ::= char\* | ''' char\* ''' | '"' char\* '"'

value ::= value<int> | value<double>

value<T> ::= T | seq<T> | 'struct' '(' seq<T> ',' ".Dim" '=' seq<int> ')'

seq<int> ::= int ':' int | cseq<int>

seq<double> ::= cseq<double>

cseq<T> ::= 'c' '(' T % ',' ')'

Definition at line 434 of file dump.hpp.

## Constructor & Destructor Documentation

|  |  |  |  |  |
| --- | --- | --- | --- | --- |
| stan::io::dump\_reader::dump\_reader | ( |  | ) |  |

inline

Construct a reader for standard input.

Definition at line 783 of file dump.hpp.

|  |  |  |  |  |  |
| --- | --- | --- | --- | --- | --- |
| stan::io::dump\_reader::dump\_reader | ( | std::istream & | *in* | ) |  |

inline

Construct a reader for the specified input stream.

Parameters
:   |  |  |
    | --- | --- |
    | in | Input stream reference from which to read. |

Definition at line 790 of file dump.hpp.

|  |  |  |  |  |
| --- | --- | --- | --- | --- |
| stan::io::dump\_reader::~dump\_reader | ( |  | ) |  |

inline

Destroy this reader.

Definition at line 795 of file dump.hpp.

## Member Function Documentation

|  |  |  |  |  |
| --- | --- | --- | --- | --- |
| std::vector<size\_t> stan::io::dump\_reader::dims | ( |  | ) |  |

inline

Return the dimensions of the most recently read variable.

Returns
:   Last dimensions.

Definition at line 813 of file dump.hpp.

|  |  |  |  |  |
| --- | --- | --- | --- | --- |
| std::vector<double> stan::io::dump\_reader::double\_values | ( |  | ) |  |

inline

Returns the floating point values from the last item if the last item read contained floating point values and the empty vector otherwise.

Returns
:   Floating point values of last item.

Definition at line 846 of file dump.hpp.

|  |  |  |  |  |
| --- | --- | --- | --- | --- |
| std::vector<int> stan::io::dump\_reader::int\_values | ( |  | ) |  |

inline

Returns the integer values from the last item if the last item read was an integer and the empty vector otherwise.

Returns
:   Integer values of last item.

Definition at line 835 of file dump.hpp.

|  |  |  |  |  |
| --- | --- | --- | --- | --- |
| bool stan::io::dump\_reader::is\_int | ( |  | ) |  |

inline

Checks if the last item read is integer.

Return `true` if the value(s) in the most recently read item are integer values and `false` if they are floating point.

Definition at line 824 of file dump.hpp.

|  |  |  |  |  |
| --- | --- | --- | --- | --- |
| std::string stan::io::dump\_reader::name | ( |  | ) |  |

inline

Return the name of the most recently read variable.

Returns
:   Name of most recently read variable.

Definition at line 803 of file dump.hpp.

|  |  |  |  |  |
| --- | --- | --- | --- | --- |
| bool stan::io::dump\_reader::next | ( |  | ) |  |

inline

Read the next value from the input stream, returning `true` if successful and `false` if no further input may be read.

Returns
:   Return `true` if a fresh variable was read.

Exceptions
:   |  |  |
    | --- | --- |
    | bad\_cast | if bad number values encountered. |

Definition at line 858 of file dump.hpp.

---

The documentation for this class was generated from the following file:

- src/stan/io/dump.hpp


---

[ Stan Home Page ]

*© 2011–2014,
Stan Development Team.*
